# Development and validation of a novel scoring system integrating MIBG scintigraphy and SPECT imaging for differentiating Parkinson’s disease

**DOI:** 10.3389/fneur.2025.1652009

**Published:** 2025-10-27

**Authors:** Pei Yin, Yong Wang, Lizhuo Jia, Tiancheng Hao, Yufei Gao, Siqi Wu, Jiangmeng Wu, Danning Wang

**Affiliations:** Department of Radiology and Nuclear Medicine, The First Hospital of Hebei Medical University, Shijiazhuang, Hebei, China

**Keywords:** Parkinson’s disease, parkinsonian syndromes, MIBG scintigraphy, SPECT, diagnostic model, scoring system, ROC curve

## Abstract

**Background:**

Early and accurate differentiation of Parkinson’s disease (PD) from other parkinsonian syndromes (PS) is crucial for appropriate management and prognostication. This study aimed to develop and evaluate a diagnostic model and a simplified scoring system combining clinical features, with parameters from cardiac ^¹³¹^I-MIBG scintigraphy, including planar-derived quantitative data (H/M ratio, clearance rate) and qualitative SPECT findings (uptake uniformity).

**Methods:**

This retrospective study included 102 patients clinically diagnosed with PD and 71 patients with PS, based on their final diagnostic classification after follow-up. Data on demographic characteristics, clinical features, MIBG scintigraphy (15-min and 4-h heart-to-mediastinum (H/M) ratios, 4-h clearance rate), and SPECT findings (categorized as no uptake, uniform, or non-uniform) were collected. Patients with missing SPECT data were excluded. Univariate analyses were performed, and variables with *p* < 0.1 were included in a multivariate logistic regression model using backward selection. A simplified scoring system was derived from the logistic model. Receiver operating characteristic (ROC) curve analysis was used to assess diagnostic performance.

**Results:**

The final logistic regression model identified 4-h H/M ratio (OR 0.109, 95% CI 0.033–0.358), 4-h clearance rate (OR 4.500, 95% CI 1.030–19.651), and SPECT findings as significant predictors of PD. The logistic model achieved an area under the ROC curve (AUC) of 0.810 (95% CI 0.744–0.876), with a sensitivity of 81.4% and specificity of 70.4%. A derived combined score (ranging from 3 to 9 points) demonstrated an AUC of 0.800 (95% CI 0.736–0.864), with a sensitivity of 78.4% and specificity of 70.4% at a cutoff of 5 points.

**Conclusion:**

A combination of MIBG scintigraphy parameters and SPECT imaging provides good diagnostic accuracy for differentiating PD from PS. The developed logistic regression model and the simplified scoring system offer promising tools for clinical practice, potentially improving diagnostic precision. Further prospective validation in larger, diverse cohorts is warranted.

## Introduction

Parkinson’s disease (PD) is the second most common neurodegenerative disorder, characterized by motor symptoms such as bradykinesia, rigidity, tremor, and postural instability (1, 2). However, the early clinical diagnosis of PD can be challenging due to overlapping symptoms with other parkinsonian syndromes (PS), including multiple system atrophy (MSA), progressive supranuclear palsy (PSP), and corticobasal degeneration (CBD) (3, 4). Accurate differentiation between PD and PS is critical as they have distinct prognoses, treatment responses, and underlying pathologies (5).

Cardiac ^131^I-metaiodobenzylguanidine (MIBG) scintigraphy, which assesses cardiac sympathetic denervation, has emerged as a valuable biomarker for PD, as cardiac sympathetic denervation is typically present in PD but often preserved in MSA, PSP, and other PS (6–8). Parameters derived from MIBG scintigraphy, such as the early and delayed heart-to-mediastinum (H/M) ratios and washout rate (WR) or clearance rate, have shown good diagnostic utility (9, 10).

While individual biomarkers offer diagnostic value, combining multiple parameters from different modalities may enhance diagnostic accuracy (11, 12). Several studies have explored combining MIBG scintigraphy with other clinical or imaging markers, but a simple, easy-to-use scoring system based on robustly selected variables is still desirable for clinical settings (13). Therefore, this study aimed to develop and evaluate a diagnostic model incorporating MIBG scintigraphy parameters and SPECT findings to differentiate PD from PS, and to subsequently derive a simplified, practical scoring system for clinical application, based on a retrospectively analyzed cohort.

## Methods

### Study design and participants

This retrospective study was conducted by reviewing medical records of patients evaluated for parkinsonism at The First Hospital of Hebei Medical University between December 2022 and January 2025. The final study cohort for statistical modeling consisted of 173 patients: 102 diagnosed with PD and 71 with PS. These diagnoses were established by experienced neurologists specializing in movement disorders, based on comprehensive clinical evaluation including motor and non-motor symptoms, response to levodopa where applicable, and longitudinal follow-up for at least 2 years to confirm diagnostic stability. Specifically, the diagnosis of PD was made according to the 2015 Movement Disorder Society (MDS) clinical diagnostic criteria for Parkinson’s disease (2), while diagnoses for PS subtypes were based on their respective international consensus criteria; specifically, Multiple System Atrophy (MSA) was diagnosed according to the Movement Disorder Society criteria (14), Progressive Supranuclear Palsy (PSP) and Corticobasal Degeneration (CBD) according to the latest consensus criteria (15), Dementia with Lewy Bodies (DLB) based on the 2017 revised criteria (16), and Vascular Parkinsonism (VaP) using established clinical-radiological criteria (17). The PS group was heterogeneous, comprising multiple system atrophy (MSA), progressive supranuclear palsy (PSP), and other related disorders, with a detailed breakdown provided in Supplementary Table S1. The “group” variable in the dataset, reflecting this final diagnostic classification, was used as the outcome for this study.

Patients were selected for the final analysis based on the availability of complete data for the variables included in the statistical model. Specifically, as per the pre-defined analysis plan (“SPECT-missing patients were excluded”), patients with missing or uninterpretable SPECT data (essential for categorization into “no uptake,” “uniform,” or “non-uniform”) were excluded from this cohort of 173 patients. The study protocol was approved by the Institutional Review Board of The First Hospital of Hebei Medical University, and the requirement for informed consent was waived due to the retrospective nature of the study and the use of de-identified data. This study adheres to the principles of the Declaration of Helsinki and relevant STARD (Standards for Reporting of Diagnostic Accuracy Studies) guidelines (18).

### Data collection

The following data were extracted from patient medical records for the analyzed cohort:

*Demographic and clinical data*: Age at examination (years), sex, disease duration (months from symptom onset to examination), UPDRS-III score, and Hoehn and Yahr (H-Y) stage. Blank entries for UPDRS-III or H-Y in the raw dataset were treated as missing for those specific entries but patients were included if other core data were present. Comorbidities such as hypertension, diabetes, and history of cerebral infarction were recorded as present or absent.

*Non-motor symptoms*: Constipation and Hyposmia (Smell Abnormality) were documented as present or absence based on patient history or clinical assessment.

131*I-MIBG scintigraphy*: Cardiac MIBG scintigraphy was performed according to a standardized institutional protocol consistent with general guidelines (9). Patients received an intravenous injection of 185 MBq of ^131^I-MIBG after thyroid blockade. Planar thoracic images were acquired at approximately 15 min (early) and 4 h (delayed) post-injection. The heart-to-mediastinum (H/M) ratio was calculated from ROIs placed on the heart and upper mediastinum. To ensure consistency, a semi-automated method was used, where ROIs were initially placed by software and subsequently reviewed and manually adjusted if necessary by an experienced nuclear medicine physician (P. Y.) blinded to the clinical diagnosis. The 4-h clearance rate was calculated as: ([Early H/M - Delayed H/M] / Early H/M) x 100%. The MIBG uptake quality was noted, and patients with non-diagnostic MIBG scans were excluded.

*SPECT imaging*: SPECT findings were qualitatively interpreted by experienced nuclear medicine physicians blinded to the final clinical diagnosis. For the purpose of this study, SPECT results were categorized as: *No uptake (Code 0)*: Severe, bilateral reduction of tracer uptake. *Uniform (Code 1)*: Generally symmetrical and relatively preserved or mildly symmetrically reduced uptake. *Non-uniform (Code 2)*: Asymmetric reduction in tracer uptake, typically more pronounced in the putamen contralateral to the more affected limb, or a clear asymmetric pattern.

### Statistical analysis

Statistical analyses were performed using SAS software, version 9.4 (SAS Institute Inc., Cary, NC, United States). Continuous variables were expressed as median and interquartile range (IQR), and categorical variables as number (n) and percentage (%). Comparisons between the PD and PS groups were made using the Wilcoxon two-sample test for continuous variables and the Chi-square test or Fisher’s exact test for categorical variables, as appropriate. A two-sided *p*-value < 0.05 was considered statistically significant.

The variable selection process was conducted in two stages. First, candidate predictors were chosen based on their established diagnostic relevance in the literature and clinical availability. These included demographic data (age), clinical metrics (disease duration, UPDRS-III, H-Y stage), key non-motor symptoms (constipation, hyposmia), and all available MIBG and SPECT imaging parameters. In the second stage, these candidate predictors were subjected to univariate analysis. Variables demonstrating a potential association with the diagnosis (defined by a *p*-value < 0.1) were then included in the multivariate logistic regression model. A backward stepwise selection method was used, with *p*-values for entry and removal set at 0.1. The model’s discrimination was assessed using the area under the ROC curve (AUC), and its calibration was assessed using the Hosmer-Lemeshow test.

Based on the final logistic regression model, a simplified integer-based scoring system was developed. Coefficients from the logistic regression model were used to guide the assignment of points for different levels or categories of the retained predictor variables, aiming for clinical ease of use (19).

The diagnostic performance of individual MIBG parameters, SPECT findings, the logistic regression model, and the derived scoring system was evaluated using receiver operating characteristic (ROC) curve analysis. The AUC with its 95% confidence interval (CI) was calculated. The optimal cutoff value was determined using Youden’s index. The DeLong test was used to compare the AUCs of different models, with the combined logistic model serving as the reference.

## Results

### Patient characteristics

A total of 173 patients formed the study cohort, comprising 102 patients with a final diagnosis of PD and 71 patients with PS. Baseline demographic and clinical characteristics of these two groups are summarized in Table 1. Patients in the PD group had a slightly longer disease duration compared to the PS group (median 29 months vs. 24 months, *p* = 0.073). Significant differences were observed in MIBG scintigraphy parameters: the PD group had significantly lower 15-min H/M ratios (*p* < 0.001) and 4-h H/M ratios (*p* < 0.001) compared to the PS group. The 4-h MIBG clearance rate was significantly different between groups (median 0.02 in PD vs. −0.08 in PS, *p* = 0.001). SPECT findings also differed significantly between the groups (*p* < 0.001), with a higher proportion of PD patients showing non-uniform uptake (72.55%) compared to PS patients (47.89%).

**Table 1:** Baseline characteristics of patients in PD and PS groups.

Variable	PD (*n* = 102)	PS (*n* = 71)	*p*-value
Age (years), median (IQR)	65 (58, 71)	67 (61, 72)	0.152
Disease Duration (months), median (IQR)	29 (17, 61)	24 (15, 37)	0.073
UPDRS-III Score, median (IQR)	26 (19, 35)	28 (21, 34)	0.662
Hoehn-Yahr Stage, median (IQR)	2 (2, 2.5)	2 (2, 2.5)	0.249
15 min H/M Ratio, median (IQR)	1.65 (1.38, 1.94)	2.07 (1.84, 2.35)	<0.001
4 h H/M Ratio, median (IQR)	1.57 (1.27, 2.02)	2.29 (1.88, 2.60)	<0.001
4 h Clearance Rate, median (IQR)	0.02 (−0.08, 0.10)	−0.08 (−0.17, 0.04)	0.001
Constipation			
No, *n* (%)	54 (52.94)	46 (64.79)	0.180
Yes, *n* (%)	48 (47.06)	25 (35.21)	
Hyposmia			
No, *n* (%)	79 (77.45)	62 (87.32)	0.100
Yes, *n* (%)	23 (22.55)	9 (12.68)	
SPECT Findings			
No uptake, *n* (%)	21 (20.59)	6 (8.45)	<0.001
Uniform, *n* (%)	7 (6.86)	31 (43.66)	
Non-uniform, *n* (%)	74 (72.55)	34 (47.89)	

PD, Parkinson’s Disease; PS, Parkinsonian Syndromes; IQR, Interquartile Range; UPDRS-III, Unified Parkinson’s Disease Rating Scale Part III; H/M, Heart-to-Mediastinum Ratio; SPECT, Single-Photon Emission Computed Tomography. *p*-values from Wilcoxon two-sample test for continuous variables and Chi-square or Fisher’s exact test for categorical variables.

### Multivariable logistic regression model

Variables with *p* < 0.1 in univariate analysis were entered into the logistic regression model. After backward selection, the final model retained 4-h H/M ratio, 4-h clearance rate, and SPECT findings as significant independent predictors of PD (Table 2). The Hosmer-Lemeshow test indicated good model calibration (*χ*^2^ = 8.12, *p* = 0.422), suggesting no significant difference between the predicted probabilities and observed outcomes. The calibration of the model is further illustrated in Supplementary Figure S1. Lower 4-h H/M ratios and non-uniform SPECT patterns were associated with an increased likelihood of a PD diagnosis. Conversely, a higher 4-h clearance rate was associated with a higher likelihood of PD.

**Table 2:** Multivariate logistic regression model parameters and odds ratios for predicting PD.

Variable	Parameter Estimate (*β*) ^a^	Standard Error ^a^	Wald Chi-Square ^a^	*p*-value ^a^	Odds Ratio (OR) ^b^	95% CI for OR ^b^
Intercept	4.185	1.124	13.870	0.000	N/A	N/A
4 h H/M Ratio (per unit increase)	−2.220	0.608	13.320	0.000	0.109	0.033–0.358
4 h Clearance Rate (per unit increase)	1.504	0.751	4.008	0.045	4.500	1.030–19.651
SPECT (Reference: Non-uniform) ^a,b^				0.003 ^a^ (Overall for SPECT variable)		
No uptake vs. Non-uniform	0.083 ^a^	0.447 ^a^	0.034 ^a^ (Wald for this level)		0.481 ^b^	0.143–1.613 ^b^
Uniform vs. Non-uniform	−0.897 ^a^	0.413 ^a^	4.721 ^a^ (Wald for this level)		0.181 ^b^	0.063–0.521 ^b^

PD, Parkinson’s Disease. ^a^Parameter estimates (β), Standard Error, Wald Chi-Square, and *p*-values are from the original logistic regression output. The reference category for SPECT is “Non-uniform.” ^b^Odds Ratios (OR) and 95% Confidence Intervals (CI) are presented for enhanced interpretability.

### Development of the combined scoring system

A simplified scoring system, termed the “Combined Score,” was developed based on the variables retained in the logistic regression model. Points were assigned to different categories or ranges of these variables to create an additive score, as detailed in Table 3. This table provides the complete scoring algorithm. The total score could range from 3 to 9 points, with higher scores designed to indicate a higher likelihood of PD.

**Table 3:** The combined scoring system for predicting PD.

Variable	Variable level	Assigned points
4-h H/M Ratio	<1.35	4
	1.35–1.84	3
	1.85–2.34	2
	≥2.35	1
4-h Clearance Rate	≥0.0	2
	<0.0	1
SPECT Findings	Non-uniform	3
	Uniform	2
	No uptake	1

Points are summed to obtain a total Combined Score (range 3–9). Higher scores suggest a higher probability of PD. Categorization and point allocation were based on logistic model coefficients and clinical applicability.

### Diagnostic performance of individual and combined predictors

**Figure 1:**
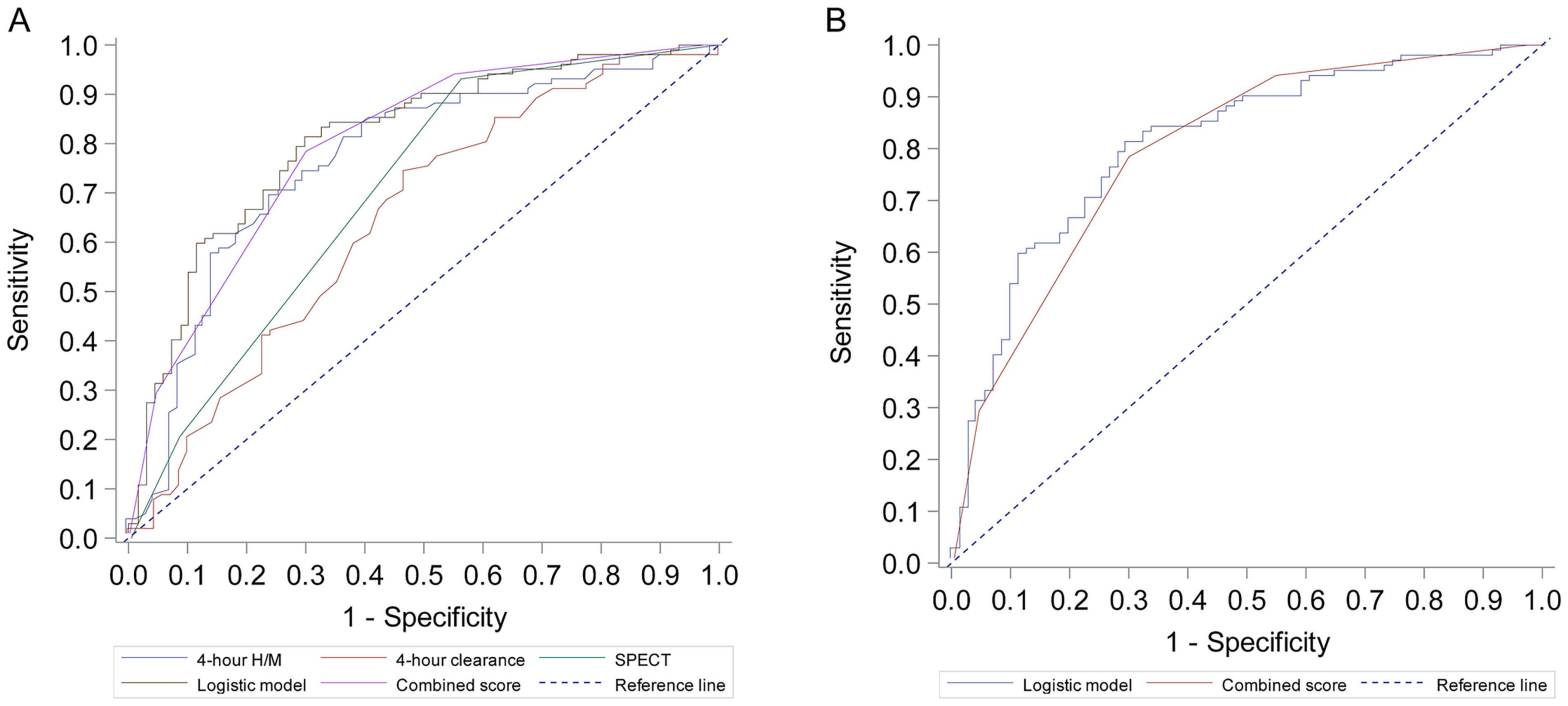
Receiver operating characteristic (ROC) curves for prediction of parkinson’s disease. **(A)** ROC curves for individual predictors: 4-h H/M ratio, 4-h clearance rate, and SPECT findings. **(B)** ROC curves for the combined multivariate logistic regression model and the derived Combined Score.

The diagnostic performance of individual parameters, the logistic regression model, and the Combined Score are detailed in Table 4 and Figure 1. The 4-h H/M ratio showed good individual predictive ability with an AUC of 0.766. The combined logistic regression model demonstrated the highest diagnostic accuracy with an AUC of 0.810 (95% CI 0.744–0.876), achieving a sensitivity of 81.4% and specificity of 70.4%. The simplified Combined Score also showed strong diagnostic performance with an AUC of 0.800 (95% CI 0.736–0.864), which was not significantly different from the logistic model’s AUC (*p* = 0.563). At an optimal cutoff value of ≥5 points, the Combined Score yielded a sensitivity of 78.4% and a specificity of 70.4%.

**Table 4:** Diagnostic performance of individual and combined predictors for PD.

Predictor	AUC	95% CI for AUC	*p*-value*	Optimal Cutoff^#^	Sensitivity (%)	Specificity (%)
4-h H/M Ratio	0.7658	0.6910–0.8406	0.025	≤1.85	69.6	76.1
4-h Clearance rate	0.6472	0.5619–0.7325	<0.001	<0.01 ^†^	74.5	53.5
SPECT findings	0.7026	0.6324–0.7729	<0.001	Non-uniform ^§^	93.1	43.7
Combined score	0.8001	0.7360–0.8643	0.563	≥5 points	78.4	70.4
Logistic model	0.8103	0.7443–0.8763	N/A	(Probability Cutoff)^α^	81.4	70.4

AUC, Area Under the ROC Curve; CI: Confidence Interval. *p-value for comparison of AUC with the logistic model’s AUC (DeLong test). ^#^Optimal cutoff determined by Youden’s index from original analysis. ^†^For 4 h Clearance Rate, PD group had median 0.02 vs PS -0.08. A cutoff of “<0.01” for a test where higher values indicate PD usually means values at or above this threshold. The sensitivity/specificity reported are taken from the original analysis. ^§^For SPECT, the high sensitivity (93.1%) and moderate specificity (43.7%) at “Non-uniform” cutoff suggests this category, possibly combined with “No uptake,” was considered indicative of PD for this specific single-predictor performance assessment. (e.g., PD cases with Non-uniform or No Uptake / Total PD = (74 + 21)/102 = 93.1%). This specific definition for SPECT as a standalone test should be explicit. ^α^The logistic model uses a probability cutoff corresponding to the maximal Youden’s index.

## Discussion

The accurate differentiation of PD from other PS remains a significant clinical challenge. This study developed and evaluated a diagnostic model and a simplified scoring system by integrating MIBG scintigraphy and its associated cardiac SPECT imaging. Our results confirm that a combination of 4-h MIBG H/M ratio, 4-h MIBG clearance rate, and SPECT imaging can effectively distinguish PD from PS, with the combined logistic model achieving an AUC of 0.810 and a derived simplified score achieving a comparable AUC of 0.800.

The individual MIBG parameters, particularly the 4-h H/M ratio (AUC 0.766), showed good discriminatory ability, consistent with previous literature highlighting cardiac sympathetic denervation as a key feature of PD (6, 8, 20). Reduced MIBG uptake in PD reflects Lewy body pathology affecting cardiac sympathetic nerves (7). The 4-h clearance rate also contributed significantly. In our cohort, PD patients tended to have higher (less negative or positive) clearance rate values compared to PS patients, potentially reflecting impaired MIBG storage or altered turnover kinetics (10, 21). We note that this finding, where a higher clearance rate is associated with PD, contrasts with some studies that define washout rate differently [as (early counts - delayed counts)/early counts], in which a higher rate also indicates abnormality (22, 23). Our definition and finding reflect an altered kinetic profile that nonetheless differentiated the groups effectively in our statistical model.

A novel aspect of our study is the integration of qualitative cardiac SPECT findings with standard planar MIBG parameters, all derived from a single scintigraphy session. The SPECT analysis provided significant, independent diagnostic information, with a “non-uniform” uptake pattern being strongly associated with PD. This suggests that the spatial heterogeneity of cardiac denervation is a key feature that helps differentiate PD from PS. Our approach enhances the diagnostic utility of a single MIBG study by extracting both quantitative data (H/M ratio, clearance rate) and qualitative spatial information (uptake pattern) without requiring an additional imaging modality or tracer (7, 24).

Our approach combines semi-quantitative MIBG analysis with a qualitative SPECT assessment, which reflects a common workflow in many clinical nuclear medicine departments. Previous studies have explored combining quantitative SPECT, using the specific binding ratio (SBR), with MIBG scintigraphy. For instance, Uyama et al. reported a diagnostic accuracy of 79.4% by combining SBR and delayed H/M ratio cutoffs (25). While quantitative SBR analysis may enhance objectivity, our logistic model achieved a slightly higher diagnostic performance (AUC of 0.810) and, critically, was used to derive a simplified scoring system. The advantage of our proposed score lies in its direct applicability without requiring specialized software for SBR calculation, potentially offering broader utility. Nevertheless, the integration of quantitative analysis represents an important future direction for refining such diagnostic models.

A key contribution of this work is the derivation of a simple, integer-based “Combined Score.” This score offers a practical tool that can facilitate diagnostic decision-making (13, 19) and shows performance comparable to other combined diagnostic approaches (11, 26–28).

This study has several limitations. First, its retrospective design may be subject to selection and information bias. Second, the sample size, while informative, warrants external validation. Third, the PS group was heterogeneous, which may influence the model’s performance on specific subtypes. Fourth, our study relied on a qualitative SPECT assessment, which, though common in clinical practice, is subject to inter-rater variability and may be less reproducible than quantitative analyses (29). Fifth, the retrospective nature of the study precluded an *a priori* sample size calculation; however, the final model was developed with 71 events in the smaller group for three main predictor variables, providing an events-per-variable ratio of approximately 23, which helps mitigate the risk of overfitting (30). Furthermore, the model and scoring system were developed and tested on the same dataset without a separate internal or external validation cohort. Techniques such as bootstrapping or cross-validation were not performed. Therefore, the reported performance may be optimistic, and the generalizability of our findings is unknown. This underscores the critical need for prospective validation in independent, multicenter cohorts before the score can be recommended for widespread clinical use. Finally, lack of widespread neuropathological confirmation, the ultimate gold standard, is a common limitation in such diagnostic studies.

uture research should focus on prospective validation of this Combined Score. Incorporation of other biomarkers like olfactory testing, REM sleep behavior disorder assessment, or alpha-synuclein seed amplification assays could further refine diagnostic accuracy, though current biomarker-based models often suffer from insufficient sample size and low events-per-predictor ratios, limiting their reliability (31).

## Conclusion

This study demonstrates that a diagnostic model combining 4-h MIBG H/M ratio, 4-h MIBG clearance rate, and cardiac SPECT imaging provides good accuracy in differentiating PD from PS. The derived “Combined Score” offers a simple tool with comparable diagnostic performance. If validated prospectively, this scoring system could aid clinicians in achieving earlier and more accurate diagnosis of PD.

## Data Availability

The original contributions presented in the study are included in the article/Supplementary material, further inquiries can be directed to the corresponding author/s.

## Supplementary material

Supplementary Figure S1 Calibration plot for the logistic regression model. The plot shows the agreement between the predicted probabilities of PD from the logistic model (x-axis) and the observed proportion of PD patients (y-axis) across deciles of risk. The dashed line represents perfect calibration. The solid line represents the model’s calibration, which closely follows the ideal line, indicating good agreement.
