# Supplementary figures and images for: Development and validation of a novel scoring system integrating MIBG scintigraphy and SPECT imaging for differentiating Parkinson’s disease
Source: Front Neurol. 2025 Oct 27;16:1652009. doi: 10.3389/fneur.2025.1652009 (PMC12597759; doi:10.3389/fneur.2025.1652009)

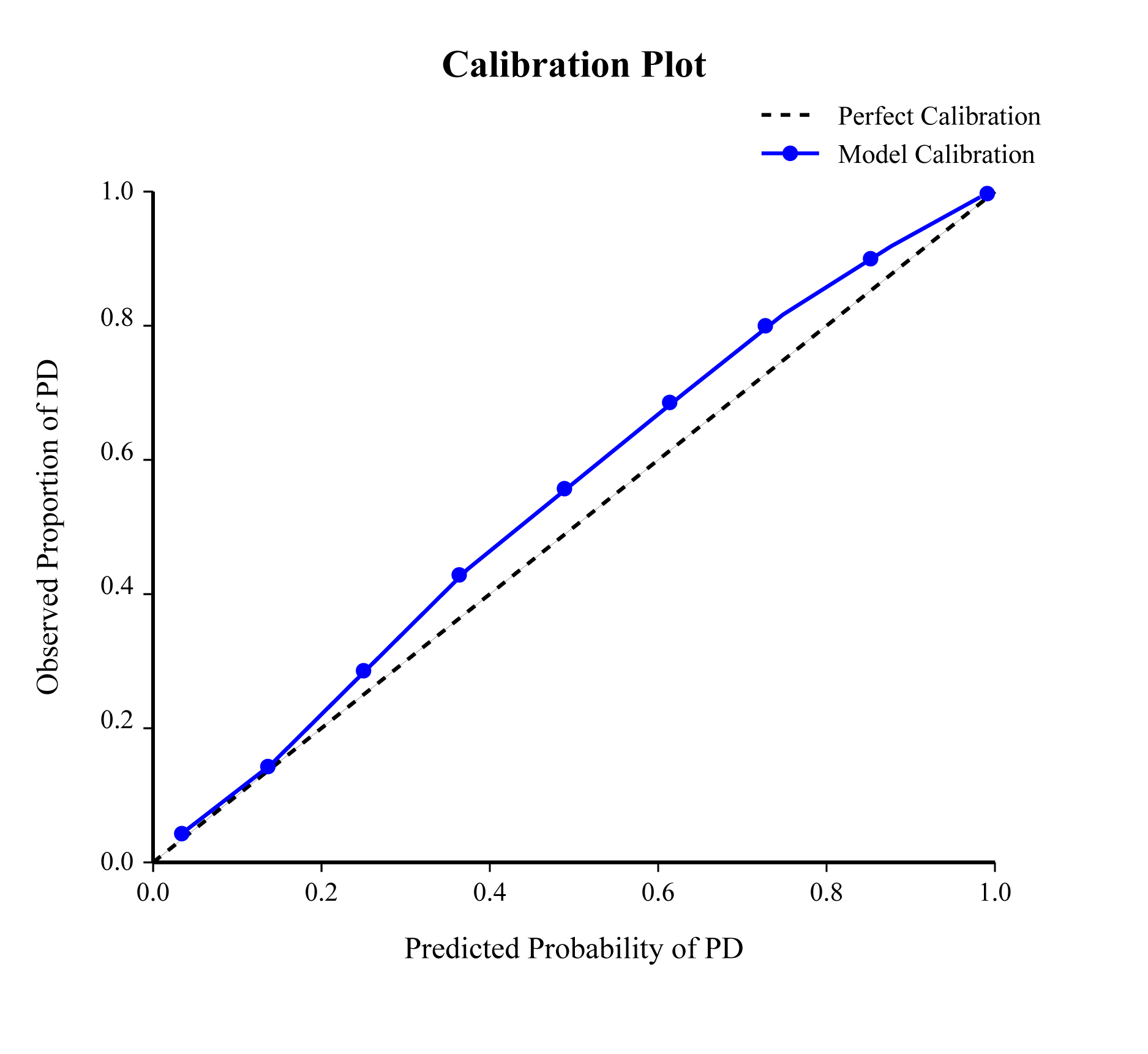

Supplement: Supplementary Figure S1 — Calibration plot for the logistic regression model. The plot shows the agreement between the predicted probabilities of PD from the logistic model (x-axis) and the observed proportion of PD patients (y-axis) across deciles of risk. The dashed line represents perfect calibration. The solid line represents the model’s calibration, which closely follows the ideal line, indicating good agreement. [file Image_1.JPEG]
