# Supplementary material for: Development and validation of a novel scoring system integrating MIBG scintigraphy and SPECT imaging for differentiating Parkinson’s disease
Source: Front Neurol. 2025 Oct 27;16:1652009. doi: 10.3389/fneur.2025.1652009 (PMC12597759; doi:10.3389/fneur.2025.1652009)
Supplement: Supplementary file 2 [file Table_1.DOCX]

**Supplementary Table S1. Breakdown of Final Clinical Diagnoses in the Parkinsonian Syndromes (PS) Group (n=71)**

| **Final Diagnosis** | **Number of Patients** | **Percentage (%)** |
| --- | --- | --- |
| Multiple System Atrophy (MSA) | 25 | 35.2 |
| Progressive Supranuclear Palsy (PSP) | 20 | 28.2 |
| Dementia with Lewy Bodies (DLB) | 12 | 16.9 |
| Corticobasal Degeneration (CBD) | 5 | 7.0 |
| Vascular Parkinsonism (VaP) | 5 | 7.0 |
| Other (e.g., Drug-induced, Essential Tremor with parkinsonian features) | 4 | 5.6 |
| **Total** | **71** | **100.0** |
